# TOWARD: a metabolic health intervention that improves food addiction and binge eating symptoms

**DOI:** 10.3389/fpsyt.2025.1612551

**Published:** 2025-07-24

**Authors:** Erin Saner, Tro Kalayjian, Laura Buchanan, Matthew Calkins, Adrian Soto-Mota, Daekwang Jun, Shebani Sethi

**Affiliations:** ^1^ Department of Family and Community Medicine, School of Medicine, Wake Forest University, Winston Salem, NC, United States; ^2^ Private Practitioner, Tappan, NY, United States; ^3^ Private Practitioner, Rural Hall, NC, United States; ^4^ Metabolic Diseases Research Unit, National Institute of Medical Sciences and Nutrition Salvador Zubiran, Mexico City, Mexico; ^5^ Tecnologico de Monterrey, School of Medicine, Mexico City, Mexico; ^6^ Metabolic Psychiatry, Stanford University School of Medicine, Stanford, CA, United States

**Keywords:** binge eating, low-carbohydrate diet, food addiction, therapeutic carbohydrate reduction, ketogenic diet

## Abstract

Food Addiction is an increasingly relevant clinical and public health issue given its impact on metabolic health, mental health, and quality of life. Therapeutic carbohydrate reduction (TCR) has been shown to improve the symptoms of food addiction as measured by the modified Yale Food Addiction Scale (mYFAS) 2.0. We discuss a novel telemedicine intervention, in an employee wellness setting, utilizing TOWARD principles: Text-based communications, Online interactions, Wellness coaching, Asynchronous education and community support, Real-time biofeedback and remote monitoring, and Dietary modification with an emphasis on TCR to treat symptoms of food addiction and binge eating. Food addiction symptoms decreased by 40.7% and binge eating symptoms decreased by 34.7%. In an employee wellness setting, we observe a metabolic health intervention can improve weight, lower financial cost through medication deprescription, and improve mental health symptoms related to food.

## Graphical Abstract

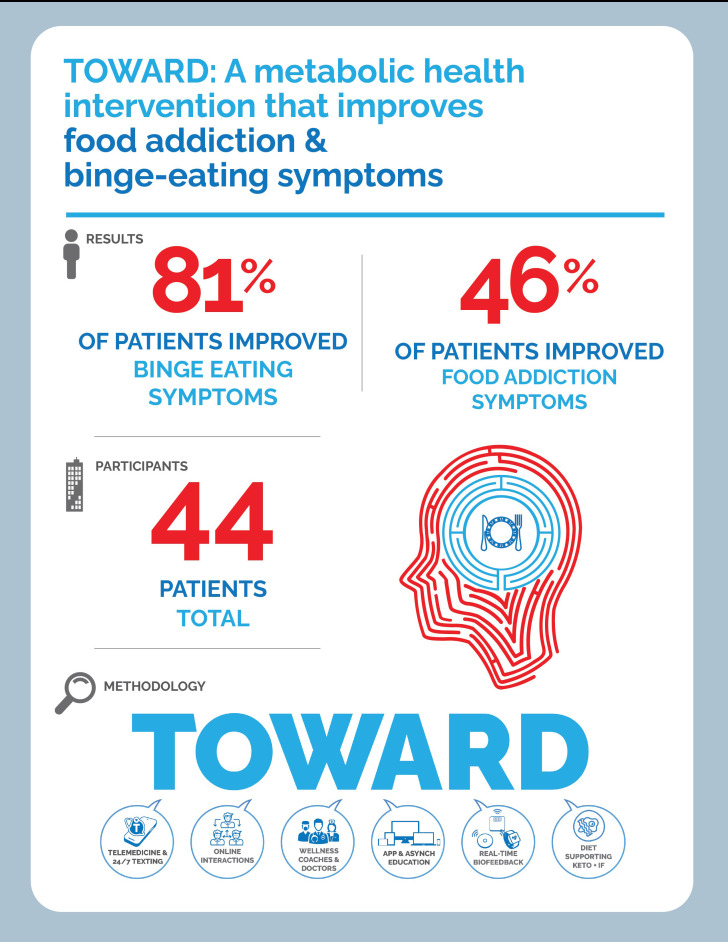

## 1 Introduction

Food addiction (FA) describes a collection of symptoms that closely mirror those observed in substance use disorders (SUD). FA is characterized by physical and psychological dependence on foods that are typically highly processed with large quantities of sugar and processed oils (1). While not formally recognized as a diagnosis in the DSM-V or ICD-10, growing research and clinical attention have focused on FA as a potential contributor to emotional distress and chronic metabolic diseases (2). The Yale Food Addiction Scale (YFAS) and its updated versions have been instrumental in characterizing addictive-like eating behaviors. The original YFAS was constructed and validated in 2009 and consisted of 25 items paralleling the DSM-IV criteria for SUD (3). This instrument has been validated across diverse populations, including non-clinical groups, individuals with obesity, and those with eating disorders. In 2017, YFAS Version 2.0 (YFAS 2.0) and mYFAS 2.0 were introduced to align with the DSM-V criteria (4). This quantitative evaluation of symptoms can be used in conjunction with other screening tools to examine the effectiveness of various clinical interventions in treating FA.

Conversely, binge eating disorder (BED) is a formal diagnosis in the DSM-V and ICD-10. Binge eating symptoms are characterized by recurrent episodes of consuming excessive amounts of food in a short period, often accompanied by a loss of control, feelings of distress, and an absence of compensatory behaviors like purging. Validated tools like the Binge Eating Scale (BES) and Binge Eating Disorder Screener 7 (BED-7) have been used to assist in determining the severity of the symptoms and aiding in diagnosis (5). Significant overlap exists between FA and BED as evidenced in a 2023 comparative review with symptoms including increased impulsivity, mood changes following food consumption, and overindulgence with difficulty controlling urges/cravings (6). There are some important differences between these two entities. FA is characterized by tolerance, withdrawal, and intense cravings for specific hyperpalatable foods, whereas BED is defined by episodic loss of control over the quantity of food consumed, often without a consistent craving for a particular type of food. These clinical entities are of increasing interest among patients and providers as they complicate the care of chronic diseases including obesity, diabetes, and mood disorders while also negatively impacting quality of life (7, 8). However, evidence suggests FA and BED are still clinically distinct entities with FA accounting for approximately 10% of the variance in binge eating behaviors and only 57% of people with binge eating behavior having clinically significant FA (9, 10).

A 2022 systematic review and meta-analysis reported an overall FA prevalence of 20%, with rates as high as 55% among individuals with BED, 28% among those with obesity, and 30% among those with type 2 diabetes (11, 12). FA impacts nearly 70 million U.S adults (13) and is a noteworthy barrier to lifestyle change and positive health outcomes (14–16). In this context, therapeutic carbohydrate reduction (TCR) using low-carbohydrate diets (LCDs) has emerged as a promising intervention. LCDs including ketogenic diets have been extensively studied in an array of patient populations including metabolic syndrome, hypertension, type 2 diabetes, migraines, Parkinson’s, epilepsy, cancer, and recently in mental health conditions (17–33). Successful application of this strategy depends on sustained behavior change. Virtual programs that combine frequent contact with education and coaching have been effective for metabolic health and may offer similar benefits in addressing food addiction (19, 22, 24). A review of the literature revealed no negative trials that employed virtual healthcare in conjunction with a low-carbohydrate approach.

Early evidence suggests similarly intensive telemedicine programs can help with addictive eating behaviors. A 2022 cohort study conducted across the UK, North America, and Sweden demonstrated promising results through combining TCR and coaching which led to meaningful decreases in FA symptom scores using the modified Yale Food Addiction Scale 2.0 (mYFAS 2.0) (34). This tracks with evidence in the SUD literature that shows intensive support groups help those with SUDs, like alcoholics anonymous for those with alcohol use disorder and 12 step facilitation (35). Treatment of metabolic disease can be challenging and often requires a multimodal approach. While pharmacotherapy and behavioral therapies are commonly cited methods for managing these conditions, TCR and intermittent fasting (IF) is an important treatment modality for consideration, particularly in the context of FA and BED (36). Additionally, pharmacologic strategies are often limited by side effects, long-term adherence, failure to address the root cause, and incomplete symptom relief, creating the need for adjunctive or alternative interventions. The primary objective of this paper is to evaluate changes in food addiction and binge eating symptoms following participation in a real-world, multimodal, metabolic health intervention incorporating TCR, IF, and remote monitoring. While the program tracked a variety of biometric, metabolic, and mental health variables, this manuscript focuses specifically on outcomes related to compulsive eating behaviors (YFAS and BES). Results related to weight, lab values, and medication deprescription were previously published (22).

## 2 Materials and methods

### 2.1 Establishment of employee metabolic health wellness program

A self-insured manufacturing company partnered with a metabolic health clinic to implement an employee metabolic wellness program in October 2021. The primary goal of the program was to holistically improve employee health, with an emphasis on weight loss and metabolic disease management as the main communication points to employees. While food addiction, binge eating assessments, and mental health were not a primary focus of recruitment or intervention, the clinical team recognized the importance of tracking compulsive eating behaviors and mental health, and integrated these assessments as part of standard patient care.

At baseline, participants completed a set of validated psychometric and metabolic health assessments, including:

- Yale Food Addiction Scale (YFAS) was initially used to evaluate food addiction symptomatology; later, the program transitioned to the modified Yale Food Addiction Scale 2.0 (mYFAS 2.0).
- Binge Eating Scale (BES) to assess the severity of binge eating behaviors
- Patient Health Questionnaire-9 (PHQ-9) as screening measure of depression symptoms

As this is a real-world, ongoing wellness program, periodic reassessment by the clinical team has informed quality improvement initiatives and led to adaptations over time. Notably, the generalized anxiety disorder 7 (GAD-7) was added as an additional psychometric tool, and the frequency of questionnaire administration increased from every two years to every six months. This led to variability in response rates and non-standardized time points for post-intervention data collection.

### 2.2 TOWARD health intervention

The TOWARD intervention employed by the metabolic health clinic is a combination of six well-defined, evidence-based best practices (22).

**Figure 1:**
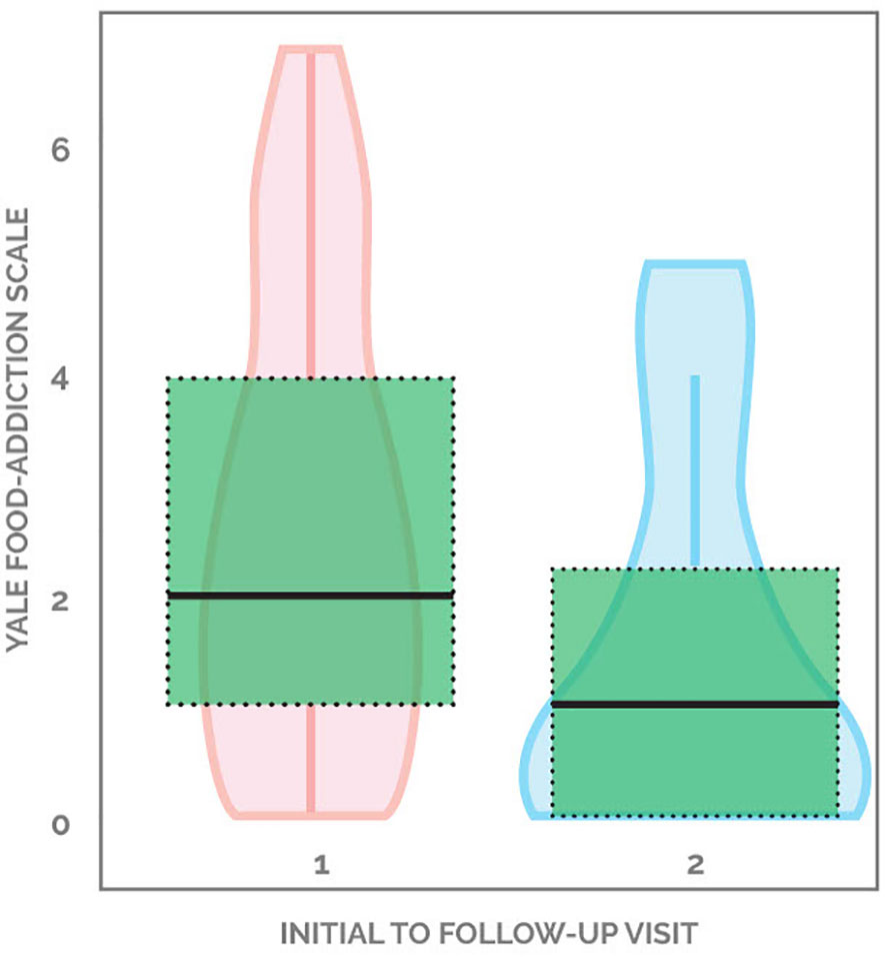
Violin plot of change in YFAS scores pre- and post-intervention.

1. Text-Based Communications & Messaging: Patients used a HIPAA-compliant text messaging system to interact with their health care team, ask questions, and receive behavioral coaching and motivational messages. Prior research has demonstrated that mobile-based interventions can improve engagement and clinical outcomes in metabolic health management.
2. Online Interactions with Clinical Teams: Participants engaged in virtual telemedicine visits with a multidisciplinary team, including physicians, physician assistants, medical assistants, personal trainers, and health coaches. These sessions provided medical oversight, individualized treatment plans, and adjustments to metabolic interventions. Visits included a combination of one-on-one sessions with patients and either their health coach or provider, as well as an initial joint visit with both the health coach and provider. See **Figure 1** from the prior publication (22).
3. Wellness Coaching: Health coaches provided structured behavioral coaching to develop strategies for reducing hyperpalatable food intake, improving satiety regulation, and managing stress-related eating.
4. Asynchronous Education & Community Support: Participants accessed an educational platform that included self-guided learning modules on the science of hunger, appetite regulation, food addiction, and emotional eating. Additionally, a monitored community group chat and company-wide webinars were used to provide additional support and reinforcement of program principles.
5. Real-Time Biofeedback & Remote Monitoring: Participants were provided with continuous glucose monitors (CGMs), glucose–ketone meters, body weight scales, and blood pressure monitors. Health coaches tracked CGM data trends, body weight fluctuations, blood pressure changes, and ketone levels via Keto Mojo meters to provide personalized recommendations and ensure safe metabolic adaptation.
6. Dietary Modifications: The program emphasized Therapeutic Carbohydrate Reduction (TCR), reducing total carbohydrates to fewer than 30g daily without counting non-starchy vegetables and leafy greens. Patients were told to focus on using avocado oil, butter, olive oil, or ghee for cooking and avoid highly processed oils and fats. Participants were encouraged to eat according to subjective hunger cues and experiment with intermittent fasting strategies tailored to their individual needs.

### 2.3 Ongoing behavioral and psychological assessments

In addition to baseline assessments, health coaches actively monitored key behavioral and psychological factors related to food addiction and binge eating:

- Self-reported data was collected weekly for the first 6 months, then monthly thereafter
- Data was gathered via one-on-one virtual coaching sessions and digital check-ins

These assessments focused on:

- Cravings intensity and frequency
- Subjective hunger levels
- Feelings of deprivation or restriction
- Social, family, and home support for dietary changes
- Emotional coping mechanisms and triggers for binge eating episodes
- Continuous glucose monitor (CGM) data trends
- Remote monitoring of body weight, blood pressure, and ketone levels using Keto Mojo meters

### 2.4 Personalized adjustments to the intervention

Health coaches used self-reported feedback and biometric data to tailor the intervention based on individual patient needs. Adjustments were made in response to:

- Increased cravings. Patients were provided with alternative foods to address their cravings with low-carb options.
- Feelings of deprivation. Patients were given both lifestyle and dietary strategies to prevent these feelings.
- Emotional stressors impacting eating behaviors. Patients were encouraged to reach out to their clinical team during stressful times.
- CGM data indicating glycemic instability
- Remote monitoring data showing weight fluctuations or blood pressure changes

As the intervention progressed, the clinical team began tracking YFAS, BES, GAD-7, and PHQ-9 scores longitudinally to monitor patient progress and ensure improvements were occurring. However, because these were not initially required components of the structured intervention, not all participants completed follow-up assessments, leading to variability in response rates and non-standardized timeframes for post-intervention data collection.

### 2.5 Statistical analysis

To enable pooled analysis, scores from both the YFAS and mYFAS 2.0 were analyzed as change from baseline instead of absolute change. Descriptive statistics were used to summarize baseline and follow-up data, including means, standard deviations, and 95% confidence intervals. To measure YFAS and BES change accounting for different follow-up times, individual variability, and testing if the change was independent from weight-loss, we used linear mixed-effect models with individual intercepts.

All analyses were conducted in R version 4.4.1

## 3 Results

Results are reported on 44 patients. 37 individuals completed two YFAS questionnaires and 37 individuals completed two BES questionnaires. 100% of individuals had a metabolic health condition and 22.7% of individuals had a baseline mental health condition. See **Table 1** for baseline characteristics.

**Table 1:** Baseline characteristics.

Baseline characteristics (n=44)	
Age (mean years ± SD)	51.1 ± 9.7
BMI (mean kg/m^2^ ± SD)	40.3 ± 7.0
BMI >40 kg/m^2^ (%)	50.0
Weight (kg ± SD)	115.4 ± 26.0
Sex	
Male (%)	36.4
Female (%)	63.6
Race	
White (%)	81.8
Black (%)	11.4
Hispanic (%)	6.8
YFAS score	2.58
BES score	17.3
Metabolic health condition (%)	100.0
Obesity (%)	97.7
T2DM or PreDM (%)	54.6
HTN (%)	54.5
Hypertriglyceridemia (%)	27.3
MASLD (%)	18.2
OSA (%)	13.6
Mental health condition (%)	22.7
Anxiety (%)	20.5
Depression (%)	13.6
ADHD (%)	2.3
History of Gastric Sleeve (%)	6.8
GLP-1 Use (%)	13.6

45.9% (17 out of 37) of patients demonstrated improvement in YFAS scores, with the average decreasing from 2.58 to 1.53, a 40.7% drop from baseline. 18.9% (seven out of 37) individuals started with a score of 0 and ended with a score of 0. 21.6% (eight out of 37) started with a non-zero score and did not change, while 13.5% (five out of 37) patients showed worsening of YFAS scores (**Figure 1**). When looking at the formal diagnosis of food addiction using the two questions evaluating clinical significance, seven started with food addiction and only two ended with food addiction (**Table 2**). **Supplementary Figure 1** demonstrates the changes in YFAS for each participant.

**Table 2:** Pre- and post-intervention prevalence of a formal diagnosis of food addiction.

Food Addiction Diagnosis	
Baseline	Follow up
None	Mild
Mild	None
Mild	None
Mild	None
Moderate	None
Moderate	None
Severe	None
Severe	Moderate

**Figure 2:**
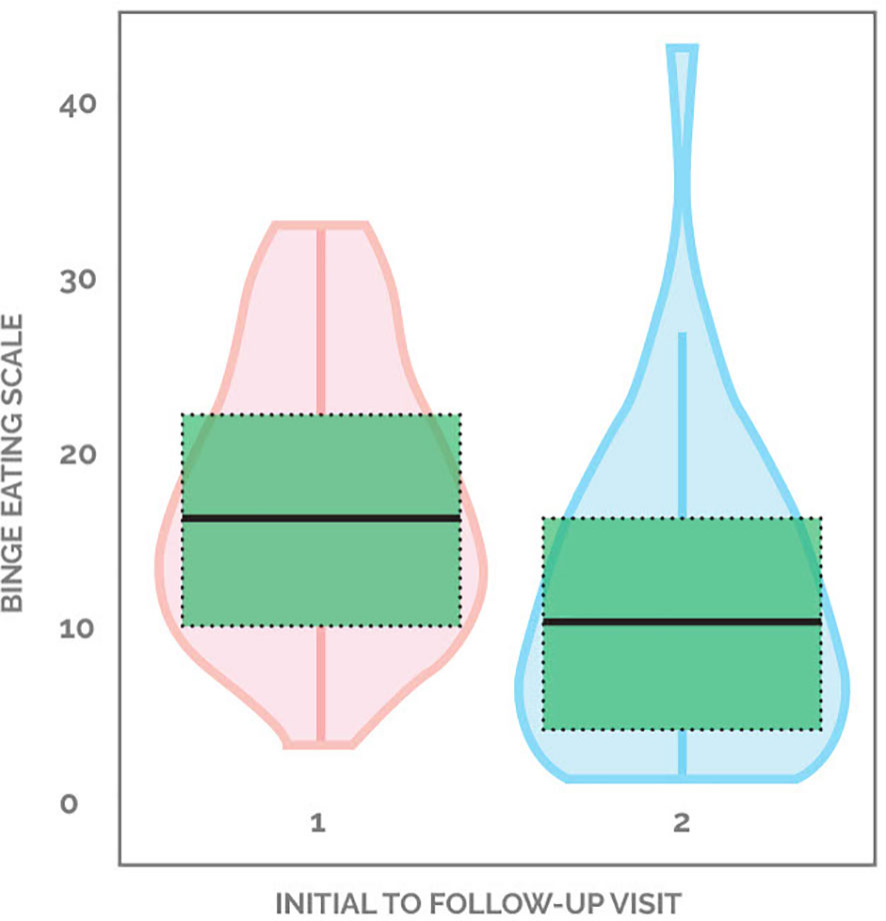
Violin plot of change in BES scores pre- and post-intervention.

81.1% (30 out of 37) of patients demonstrated an improvement in binge eating scores, with the average decreasing from 17.3 down to 11.3, a 34.7% drop from baseline. 2.7% (one out of 37) started with a non-zero score and did not change, while 16.2% (six out of 37) patients showed worsening of BES scores (**Figure 2**). **Supplementary Figure 2** demonstrates the changes in BES for each participant.

## 4 Discussion

The public health sector continues to be plagued by many chronic diseases often marked by poor metabolic and mental health. FA, particularly to ultra-processed foods, can be a significant driver of maladaptive behaviors toward progressively worsening symptoms (37). Unwin et al. (2022) utilized a whole food, low-carbohydrate diet in conjunction with intensive educational intervention using a FA recovery model (34). This ultimately led to significant reductions in patients’ FA symptoms and improved mental wellbeing (34). A case series involving three patients with obesity, binge eating, and food addiction showed remarkable improvements following a ketogenic diet (36). The current TOWARD study cohort was involved in an intensive clinical intervention using TCR, alongside remote monitoring, a smart phone application, and behavioral support, to improve FA and binge eating symptoms. Along with improvements in FA and binge eating symptoms, the TOWARD approach has been shown to improve cardiovascular risk scores, weight, while deprescribing medications (22, 38).

Recently, there has been an increased awareness of FA and the need for effective interventions. Both lifestyle modifications and medications have been studied. Lifestyle modification interventions involving intensive caloric restriction with meal replacements and gradual reintroduction of conventional foods has demonstrated clinically insignificant effects on FA symptoms (39). For example, in one study of 138 individuals, participants underwent a structured lifestyle intervention consisting of 14 weekly sessions of group counseling, caloric restriction (1000–1200 kcal/day) from a meal replacement program, and progressive physical activity up to 175 minutes per week (39). Original YFAS scores decreased from 2.24 at baseline to 1.93 post-treatment, only 13.8%. Comparatively, the TOWARD intervention showed weight-loss-independent reductions in FA symptoms by 40.7% (YFAS change of -1.12, SE: 0.39, p=0.007). Additionally, the TOWARD program did not rely on caloric restriction or meal replacements, instead emphasizing long-term dietary changes through TCR and behavioral support. In combination with the data from Unwin et al. (2022), these findings highlight that sustainable dietary modifications combined with behavioral strategies may lead to greater and potentially more sustainable improvements in FA symptoms compared to calorie-focused meal replacement interventions as recommended by supporters of the energy balance model of obesity.

Another population that has been found to have higher rates of FA is individuals undergoing bariatric surgery (40). There have been several studies showing that bariatric surgery improves FA symptoms (41, 42). Murray et al. (43) found that 16 individuals who underwent Roux-en-Y gastric bypass or sleeve gastrectomy had significant reductions in YFAS scores of approximately 1.9 to 0.9 between baseline and 4-month follow-up, whereas those receiving dietary interventions consisting of liquid meal replacements for 3 months or no treatment showed no significant changes. A larger study of 178 patients undergoing bariatric surgery found even more significant reductions in YFAS scores from 3.76 down to 2.06 at 1-year follow-up (45.2%). (41). Interestingly, there was no correlation between the reduction in food addiction symptoms and the magnitude of weight loss, a finding also observed in the TOWARD intervention. The similar magnitude of improvement in YFAS with TOWARD may offer a valuable alternative for patients seeking non-invasive, low-risk treatment options for their food addiction.

Pharmacological treatments have been explored for the management of binge eating symptoms as well as FA, such as lisdexamfetamine (LDX), semaglutide, topiramate (TPM), and other anti-obesity medications (OAOMs). Unfortunately, they come with adverse effects and are often discontinued due to these adverse effects (42, 44). Currently, LDX is the only FDA approved medication for binge eating disorder. In two studies, LDX alone significantly improved binge eating symptoms, and a combination of LDX plus TPM was also effective (42, 45). These results indicate both medications can be effective, however, their benefits must be weighed against the risks of adverse reactions and high discontinuation rates. 81.3% and 84.4% of individuals on LDX and LDX plus TPM had dry mouth, 56.3% and 20% had insomnia, 25% and 15.9% had anxiety, 18.8% and 5.7% had irritability, 25% and 11.1% had headache, and 0% and 22.2% had paresthesias, respectively (42). Other side effects with a greater than 10% occurrence rate included palpitations, bruxism, nausea, emotional liability, fatigue, ataxia, dizziness, and increase in systolic blood pressure >10 (42). In contrast, most individuals in the TOWARD program did not suffer from major side effects. A few individuals had constipation and muscle cramps that subsequently resolved with electrolyte and magnesium supplementation.

Semaglutide (SEMA) and OAOM have also shown effectiveness in improving the BES. Richards et al. found that BES decreased most substantially with a combination of SEMA and OAOM, nearly as effective with SEMA alone, and about half as effective with OAOM alone (BES reductions 8.8, 7.9, and 4.8, respectively) (46). Similarly to LDX and TPM, SEMA causes many side effects that lead to medication discontinuation rates ranging from 37-81% (47).

These findings from SEMA and OAOM are similar to our cohort that showed an improvement in BES by 6.0 points. Other pharmaceuticals have also been studied for BED. In a randomized, placebo-controlled crossover trial, phentermine-topiramate extended release was found to significantly decrease the number of binge eating days over four weeks from 16.2 days to 4.2 days, compared to 13.2 days with placebo (48). Further research is needed to evaluate the impact of the TOWARD approach on the number of binge eating days over four weeks.

Research is ongoing to explore pharmacotherapy for FA with and without binge eating. Carbone et al. (2021) found that those with binge eating disorder (BED) and FA symptoms had more severe food addiction symptoms and experienced a 47.7% drop with the use of naltrexone-bupropion (YFAS-2.0 from 6.5 to 3.4) (49). Utilizing the TOWARD intervention, 45.9% (17 out of 37) of patients demonstrated improvement in YFAS scores, with the average decreasing from 2.58 to 1.53, a 40.7% drop from baseline. These findings suggest that a structured metabolic health intervention with TCR can yield comparable reductions in binge eating symptoms and FA with comparable efficacy to pharmacotherapy, without the cost and adverse events.

Another study evaluating individuals with BED targeted inhibitory control through a cognitive training program using an antisaccade paradigm, which was found to be ineffective for food addiction symptoms (50). Another method that has been researched to improve binge eating behaviors includes cognitive behavioral therapy (CBT). CBT, both pre- and post-bariatric surgery, also effectively reduces BES scores, however, to a lesser extent than the TOWARD approach or medications. Pre-surgery tele-CBT interventions have demonstrated a 2.9 point drop in BES scores and a post-surgery tele-CBT intervention had a 4.54 drop in scores (51, 52). Future research could include evaluating medication and the TOWARD approach for treating binge eating behaviors pre- and post-bariatric surgery.

While weight-independent improvements in food addiction and binge eating symptoms were observed, additional mechanisms may contribute to these benefits. Studies have shown that intake of high-glycemic-index carbohydrates can trigger addictive symptoms (53). The reduction of high-glycemic carbohydrates may be one of the mechanisms by which TCR improves FA symptoms as has been demonstrated in multiple studies. Moreover, the behavioral coaching and community support components of the intervention may have improved emotional regulation, self-efficacy, and social connectedness, all of which could influence disordered eating patterns (54).

While many participants experienced improvement, 13.5% (5/37) had worsening of food addiction symptoms and 16.2% (6/37) experienced worsening binge eating symptoms according to the designated scales. These cases underscore the potential need for additional therapeutic support for some individuals and/or a modified approach. Clinically, acute stressors including personal, interpersonal, and occupational, seemed to cause symptom exacerbation in this cohort. One noteworthy social determinant impacting this particular cohort was geographic location in areas prone to hurricanes and climate emergencies, which we suspect contributed to reported distress symptoms. Though no participants discontinued the program due to adverse effects, some reported difficulty maintaining dietary adherence in social situations and particularly under various acute stressors.

This study has several important limitations. First, the sample size was relatively small (n=44), and incomplete follow-up data limited the ability to conduct full longitudinal analysis across all participants. The digital infrastructure may have posed barriers for individuals with limited technological proficiency or access. Additionally, self-selection bias is a possible confounder as individuals who chose to engage in the program may differ overall from those who chose not to participate or discontinued the program. Next, as the study was conducted in an employee wellness setting among insured adults, the findings may not be generalizable to unemployed, uninsured, or adolescent populations. Finally, the absence of a control group restricts our ability to infer causality between the intervention and observed outcomes. While it is well recognized that the clinical efficacy of research interventions often diminishes in real-world settings (55), this study demonstrates a key strength in being conducted within a real-world context, which enhances its external validity.

In conclusion, in an employee wellness cohort, survey respondents demonstrated improvements in FA and binge eating symptoms, highlighting the potential application of the TOWARD approach including TCR in treating food addiction and binge eating symptoms. While pharmacologic treatments such as LDX, TPM, and SEMA provide viable options for symptom management, they come with notable risks, including significant adverse effects and high discontinuation rates. Further research is warranted to explore long-term outcomes and compare head-to-head efficacy between dietary interventions, cognitive-behavioral therapy, and pharmacotherapy, as well as in different populations such as bariatric surgery recipients.

## Data Availability

The raw data supporting the conclusions of this article will be made available by the authors, without undue reservation.
