# Supplementary material for: TOWARD: a metabolic health intervention that improves food addiction and binge eating symptoms
Source: Front Psychiatry. 2025 Jul 24;16:1612551. doi: 10.3389/fpsyt.2025.1612551 (PMC12329587; doi:10.3389/fpsyt.2025.1612551)

**Supplementary Figure 1.** Changes in YFAS for each participant.
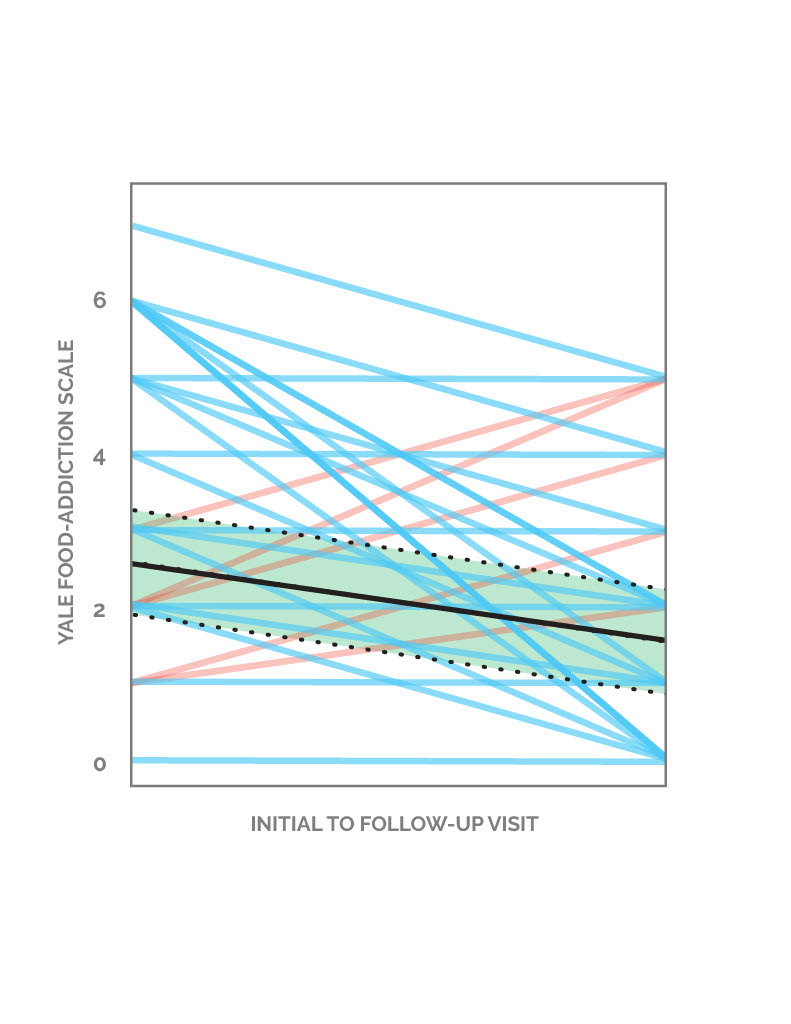


**Supplementary Figure 2.** Changes in BES for each participant.
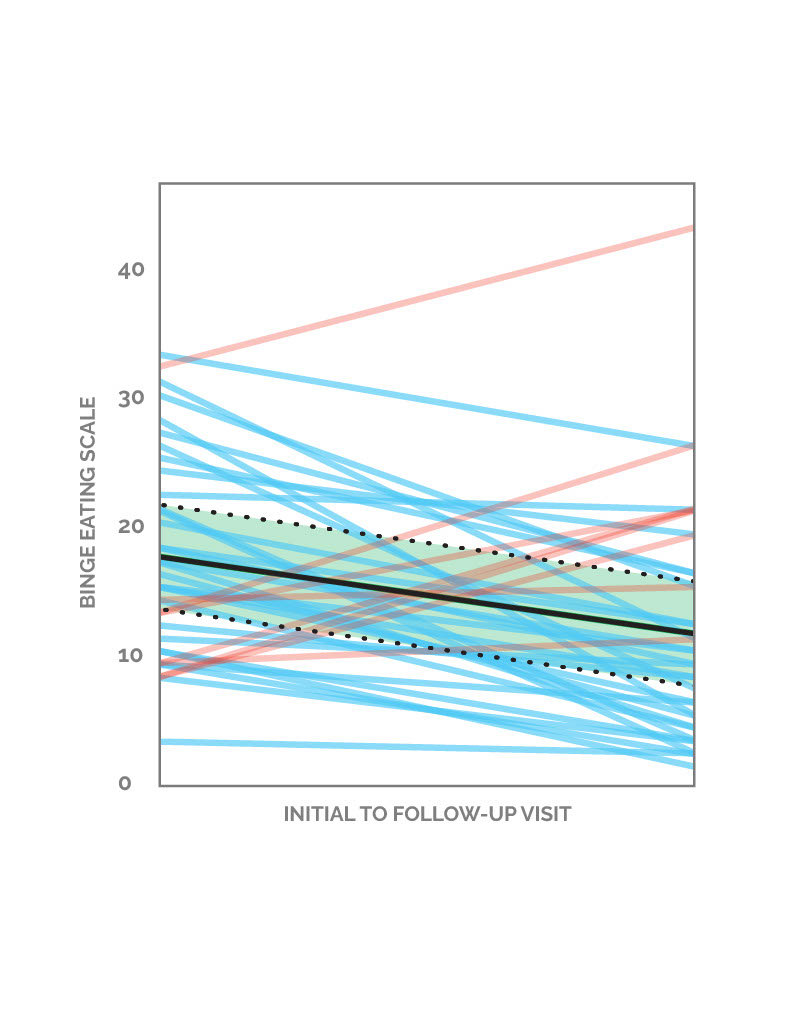

Supplement: Supplementary file 1 [file SupplementaryFile1.docx]
